# Efficacy of Polyacrylic Acid as a Conditioning Agent on the Bond Strength of Self-adhesive Resin Cements to Dental Enamel

**DOI:** 10.3290/j.ohpd.a45078

**Published:** 2020-09-04

**Authors:** Daniela Alvim Chrisostomo, Henrico Badaoui Strazzi-Sahyon, André Luiz Fraga Briso, Paulo Henrique dos Santos

**Affiliations:** a MS Student, Department of Dental Materials and Prosthodontics, Araçatuba School of Dentistry, São Paulo State University – UNESP, Araçatuba, SP, Brazil. Performed the experiments, wrote the manuscript.; b PhD Student, Department of Dental Materials and Prosthodontics, Araçatuba School of Dentistry, São Paulo State University – UNESP, Araçatuba, SP, Brazil. Contributed to the idea, hypothesis and experimental design, performed the experiments, wrote and proofread the manuscript.; c Associate Professor, Department of Restorative Dentistry, Araçatuba School of Dentistry, São Paulo State Universityy – UNESP, Araçatuba, SP, Brazil. Contributed to the idea, hypothesis and experimental design.; d Associate Professor, Department of Dental Materials and Prosthodontics, Araçatuba School of Dentistry, São Paulo State University – UNESP, Araçatuba, SP, Brazil. Contributed to the idea, hypothesis and experimental design, performed the statistical analysis, wrote and proofread the manuscript.

**Keywords:** adhesives, dental enamel, microscopy, microtensile bond strength test, resin cements

## Abstract

**Purpose:**

This in vitro study evaluated the effectiveness of polyacrylic acid as an acid etchant similar to phosphoric acid and its effect on the microtensile bond strength of self-adhesive resin cement to enamel.

**Materials and Methods:**

Ninety Te-Econom Plus resin blocks (11 x 4 mm) were cemented onto bovine enamel and distributed into 10 groups according to the surface treatments (no surface treatment; etching with 37% phosphoric acid; etching with 20% polyacrylic acid; etching with 37% phosphoric acid + dental adhesive, and etching with 20% polyacrylic acid + dental adhesive) and the self-adhesive resin cements used (RelyX U200 and MaxCem Elite) (n = 9). After bonding, the specimens were sectioned into sticks, subjected to thermocycling (5760 cycles, 5°C and 55°C) and microtensile bond strength testing (n = 6). Images of representative specimens were obtained using a scanning electron microscope. Enamel penetration evaluation of different surface treatments was analysed by confocal laser scanning microscopy (n = 3). Data on bond strength were subjected to 2-way ANOVA and Tukey’s least significant difference test (α = 0.05).

**Results:**

Both 37% phosphoric acid and 20% polyacrylic acid yielded the same microtensile bond strength between self-adhesive resin cement and enamel, independent of the application of dental adhesives (p > 0.05). MaxCem Elite showed higher bond strength values than RelyX U200 just for the 20% polyacrylic acid group (p = 0.001).

**Conclusion:**

Acid pre-conditioning of dental enamel may influence the bond strength of self-adhesive resin cement to enamel, and 20% polyacrylic acid showed efficacy similar to that of 37% phosphoric acid.

The use of self-adhesive resin cements provide less possibility of operator failure, since they simplify the adhesive luting procedures by reducing the number of steps involved,^4,40^ reducing technique sensitivity, and making the luting process simpler and faster.^1,2,15,34,35,47^ However, studies have reported a low bond strength of self-adhesive resin cement to dentin due to the limited capacity of these materials to properly etch tooth substrates.^17^ Therefore, the authors proposed prior conditioning of the dentin with materials such as polyacrylic acid,^31,45,47^ which have demonstrated satisfactory results in dentin bond strength,^30^ in order to improve its adhesion.

Self-adhesive resin cement presents difficulty in demineralization of hard tissues, such as the dental enamel.^13,21,2,7,39^ In these cases, the use of an acid conditioning agent such as phosphoric acid could provide satisfactory bond strength to enamel surface.^13,26,44^ However, if phosphoric acid comes into contact with dentin during its clinical application, it could cause deep demineralization that jeopardises the complete resin monomers infiltration, resulting in a weaker and unprotected demineralised dentin zone formation at the base of this hybrid layer promoting the deterioration over time.^10,13,20^ Other conditioning agents, such as polyacrylic acid, are widely used in restorative dentistry to prepare the dentin substrate to incorporate the glass ionomer cement.^25,29,37^ Additionally, it has been shown to yield satisfactory bond strength between self-adhesive resin cement and the dentin substrate.^30^ If this effect can be replicated on the enamel surface, a simplified and more effective adhesion protocol could be adopted.

In this sense, the purpose of this in vitro study was to evaluate the bond strength between self-adhesive resin cement and dental enamel subjected to different surface treatments. The null hypotheses tested were that the different surface treatments would not cause differences in the bond strength between the self-adhesive resin cements and enamel; and that different self-adhesive resin cements would not result in differences in the bond strength values.

## Materials and Methods

### Specimen Preparation

The materials used in this study are described in Table 1. This study was approved by the local Ethics Committee (#00317-2016).

**Table 1:** Materials, classification, composition, and batch number of materials tested

Material	Classification	Composition	Batch
Te-Econom Plus (Ivoclar Vivadent)	Hybrid resin composite	Bis-GMA, bis-EMA, UDMA, silica	R43515
RelyX U200 (3M Oral Care)	Self-adhesive resin cement	Base: glass fiber, methacrylate phosphoric acid esters, triethylene glycol dimethacrylate, silane-treated silica, sodium persulfate Catalyst: glass fiber, substitute dimethacrylate, silane-treated silica, sodium toluenesulfonate, calcium	1711000201
MaxCem Elite (Kerr)	Self-adhesive resin cement	GPDM, co-monomers (mono-, di-, and tri-functional), proprietary self-curing redox activator, methacrylate monomers, water, acetone, ethanol, inert minerals and ytterbium fluoride	6026258
Single Bond Universal (3M Oral Care)	Multimode adhesive	MDP, bis-GMA, HEMA, photoinitiators, dimethacrylate, water, ethanol, silane	639416
OptiBond All-In-One (Kerr)	Self-Etch adhesive	Acetone, ethyl alcohol, uncured methacrylate ester monomers, GPDM, inert mineral fillers, ytterbium fluoride, photoinitiators, accelerators, stabilisers, water	6166766

Bis-GMA: bisphenol-A glycidyl methacrylate; bis-EMA: ethoxylatedbisphenol-A-dimethacrylate; UDMA: urethane dimethacrylate; GPDM: glycerophosphate dimethacrylate; MDP: 10-methacryloyloxydecyl dihydrogen phosphate HEMA: 2-hydroxyethyl methacrylate.

Ninety C3 shade resin blocks (Te-Econom Plus, Ivoclar Vivadent; Schaan, Liechtenstein) were made using a metallic matrix (11 mm in diameter and 4 mm thick). Two 2-mm increments of resin composite were inserted into the matrix using a Thompson spatula, and each increment was polymerised using a polywave unit (Valo, Ultradent; South Jordan, UT, USA), for 30 s. The light intensity of the light-curing unit was 1582 mW/cm^2^, measured by radiometer (Ecel RD7, Dabi Atlante; Ribeirao Preto, SP, Brazil). The last resin increment was covered with a transparent polyester film strip and a glass microscope slide in order to flatten the resin composite and to prevent the formation of bubbles. The resin specimens were flattened with a 600-grit silicon carbide paper (Extec; Enfield, CT, USA) under water cooling using an automatic polishing machine (Aropol, Arotec, Cotia, SP, Brazil). The blocks were then sandblasted with 50-μm aluminum oxide for 5 s at a distance of 10 mm from the airborne-particle abrasion device with 4 kg/cm^2^ pressure,^38^ and cleaned using an ultrasonic unit (Cristofoli, Campo Mourao, PR, Brazil) for 5 min, and dried with an air jet.

Ninety bovine teeth were used and all teeth that exhibited excessive wear of the incisal third, cracks or fractures were excluded from this study. The selected teeth were cleaned mechanically with periodontal curettes and received prophylaxis with pumice and water. The anatomic crowns were separated from the roots 1.0 mm from the cementum-enamel junction through a transversal section with a low-speed diamond saw under water cooling using a cutter machine (Isomet 1000, Buehler; Lake Bluff, IL, USA). Subsequently, the crowns were fixed on a device attached to a drill platform bench (FGC16; Ferrari; Cotia, SP, Brazil), and cylinders of enamel (12 mm) were obtained from the middle third of the buccal surface with the aid of a diamond glass-cutting tip (12 mm in diameter, Dinser Diamond; São Paulo, SP, Brazil) under constant irrigation. The enamel specimens were flattened with 600-grit silicon carbide paper (Extec). The nonexposure of dentin substrate was verified by a stereomicroscope at magnifications of 6X and 66X (Stemi SV11, Carl Zeiss; Jena, Germany). The specimens were divided into 10 groups according surface treatments and self-adhesive resin cements (n = 9).

The enamel of specimens in the CG/U200 group did not receive any acid pre-conditioning treatment. The base paste and catalyst of translucent shade self-adhesive resin cement (RelyX U200, 3M Oral Care; St Paul, MN, USA) were mixed and applied on the resin surface, and the restoration was positioned on the dental substrate. Prior to the photoactivation process of the adhesive interface, a load of 4.9 N was placed on the assembly in order to standardise the thickness of the resin cement. Excess cement was removed using a microbrush and each side of the assembly restoration was polymerized using a Valo polywave unit (Ultradent; South Jordan, UT, USA) for 30 s. CG/Max group specimens were treated as described for the CG/U200 group. However, the transparent shade of self-adhesive resin cement was used (MaxCem Elite, Kerr; Orange, CA, USA).

The FA/U200 enamel specimens were etched using 37% phosphoric acid (FGM, Joinville, Santa Catarina, Brazil) for 30 s, washed with deionised water, and dried with air jets. The luting process was carried out as described for the CG/U200 group. The FA/Max specimens were then treated as described for the FA/U200 group; however, the MaxCem Elite self-adhesive resin cement was used.

PA/U200 group enamel specimens were conditioned using 20% polyacrylic acid (Cavity Conditioner, GC; Tokyo, Japan). The polyacrylic acid was actively applied using a microbrush on the enamel surface for 10 s, and, according to manufacturer’s recommendations, washed with deionised water, then dried with an air jet. The luting procedure was realised as described for the CG/U200 group. The PA/Max group specimens were treated as described for the PA/U200 group. However, the MaxCem Elite self-adhesive resin cement was used instead.

FA/SBU/U200 group specimens were treated as described for the FA/U200 group. However, prior to the luting procedure, a layer of dental adhesive (Single Bond Universal; 3M Oral Care) was actively applied for 20 s and dried with an air jet for 5 s. The adhesive was activated using the Valo polywave LED for 10 s. The FA/OB/Max group specimens were treated as described for the FA/SBU/U200 group. However, the OptiBond All-In-One dental adhesive (Kerr) and MaxCem Elite self-adhesive resin cement (Kerr) were used.

PA/SBU/U200 group specimens were treated as described for the PA/U200 group. However, before the luting procedure, a layer of dental adhesive was applied as described for the FA/SBU/U200 group. The PA/OB/Max group specimens were treated as described for the PA/SBU/U200 group. However, OptiBond All-In-One dental adhesive (Kerr) and MaxCem Elite self-adhesive resin cement (Kerr) were used.

After bonding, all specimens were stored in distilled water at 37°C for 24 h.^28^ After this period, sixty specimens (n = 6) were sectioned perpendicular to the adhesive-tooth interface using a low-speed diamond saw under water cooling in a cutting machine (Isomet 1000, Buehler; Lake Bluff, IL, USA) to obtain sticks with an adhesive area of approximately 1.0 mm^2^.^5,19,32,41^ It was stipulated that 6 sticks from the middle region for each specimen would be obtained, totaling 36 sticks for each experimental group. The sticks were submitted to aging by thermocycling (5°C and 55°C, 5760 cycles, 30 s) in a thermocycling machine (MSTC-3 Plus, ElQuip; São Carlos, São Paulo, Brazil).^7^

### Microtensile Bond Strength Assessment

After thermocycling, the sticks were individually submitted to microtensile testing (OM 100, Odeme Dental Research; Luzerna, SC, Brazil).^11^ The specimens were fixed with a cyanoacrylate adhesive (Loctite Super Bond Gel, Henkel, Dusseldorf, Germany) to a metallic stub and subjected to to microtensile testing at a crosshead speed of 0.7 mm/min until rupture. The bond strength values of the groups were calculated in MPa, according the formula:^41^

Ru = F / A,

where Ru is bond strength (MPa), F is the maximum force (N), and the A is the area of the adhesive interface (mm^2^), which was measured with digital caliper (Mitutoyo; Kawasaki, Japan). A value of zero was assigned to sticks that fractured before the test.

### Scanning Electron Microscopy

The fractured sticks were examined under a stereomicroscope at magnifications of 6X and 66X to analyze the failure mode.^9,12^ Failure modes were classified into four types: adhesive failure, enamel cohesive failure, resin composite cohesive failure, and mixed failure. Representative specimens were submitted to sputter coating with gold (Baltec SCD 050; Balzers, Liechtenstein) and qualitatively analysed using scanning electron microscopy (SEM-JSM5600LV, JEOL; Tokyo, Japan) to exemplify the fracture patterns.^41^

### Statistical Analysis

Data were submitted to a normality test (Shapiro-Wilk) and bond strengths were analyzed by 2-way ANOVA and Tukey’s least significant difference test (α = 0.05).

### Confocal Laser Scanning Microscopy

Thirty teeth were used for confocal laser scanning microscopy (CLSM) (n = 3). Rhodamine B was incorporated into the self-adhesive resin cements (16 μg/g) and dental adhesives (26.5 μg/ml).^6,14,16^ The flattened disks of enamel were submersed in distilled water containing fluorescein diacetate (FDA, Sigma; St Louis, MO, USA) (0.1%) for 4 h in order to promote the penetration of the dye into the enamel hydroxyapatite crystals.^18^ Subsequently, the enamel specimens were dried with an air jet and the restorative procedure was performed as described for the microtensile bond strength analysis. Using a cutting machine (Isomet 1000,

Buehler), each specimen was sliced to obtain three middle slices, which were kept in Hanks solution to maintain the pH and avoid ion loss.^3^ The analysis was performed using CLSM (Leica TCS SP2, Leica Microsystems; Wetzlar, Germany), with an argon laser at 488 nm and He-Ne laser at 453 nm providing excitation energies. The CLSM images were obtained and recorded in the fluorescent mode with an oil immersion objective lens (40X, numerical aperture 1.25).^16^ Images were recorded from three regions along the bonded interface of each specimen. CLSM images were performed with 1-μm z-step to optically section the samples to a depth up to 20 μm below the surface.^18^ This evaluation was observational and qualitative, so no statistical analysis was performed.^13^ In CLSM analysis, only visual differences between the experimental groups were considered as findings.

## Results

**Fig 1:**
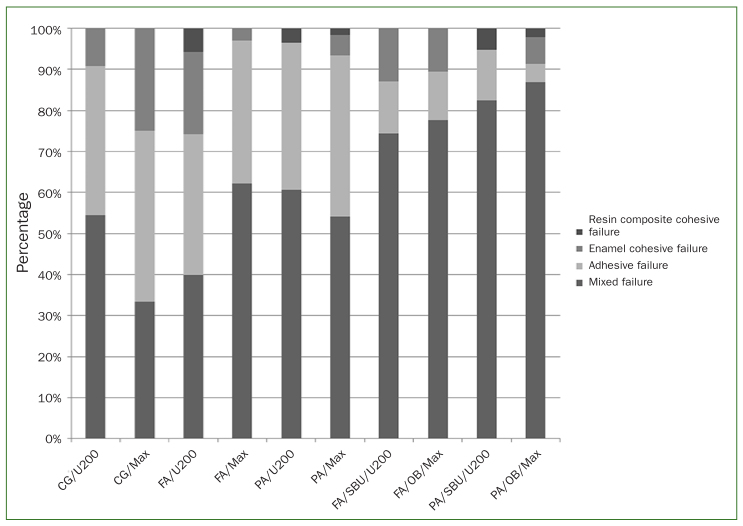
Incidence of fracture patterns (percentage) according to type of failure as function of enamel surface treatment and self-adhesive resin cement.

**Fig 2:**
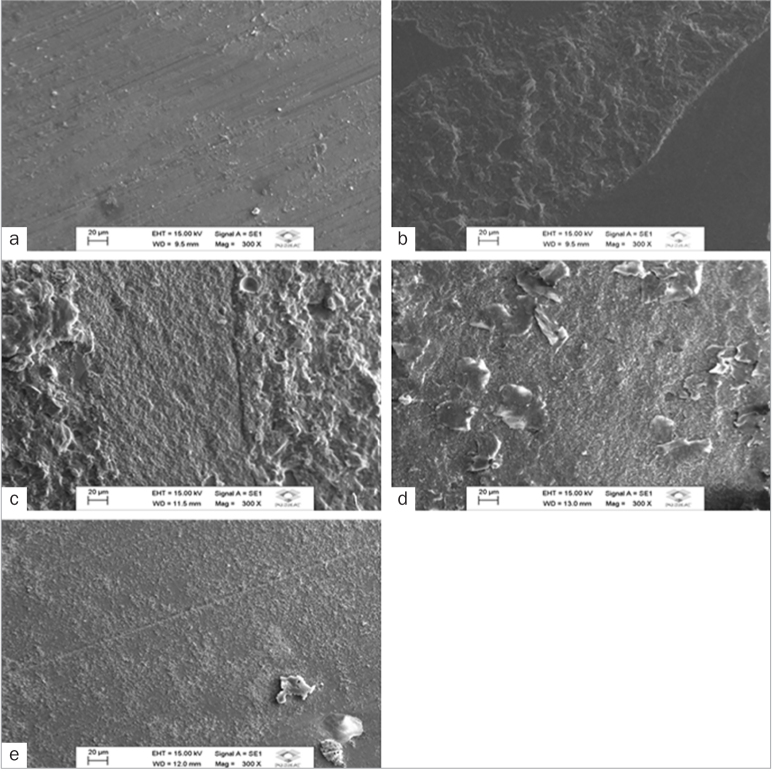
Scanning electron micrographs of representative specimens (original magnification 300X). a. Adhesive failure of dental enamel with no surface treatment luted with RelyX U200 self-adhesive resin cement (CG/U200 group). b. Mixed failure of dental enamel etched with 37% phosphoric acid (FA/U200 group). c. Mixed failure of dental enamel etched with 20% polyacrylic acid (PA/U200 group). d. Mixed failure of dental enamel etched with 37% phosphoric acid and luted with Single Bond Universal dental adhesive and RelyX U200 self-adhesive resin cement (FA/SBU/U200 group). e. Mixed failure of dental enamel etched with 20% polyacrylic acid and luted with Single Bond Universal dental adhesive and RelyX U200 self-adhesive resin cement (PA/SBU/U200 group). Little or no resin cement was observed on enamel surfaces with no surface conditioning, characterizing the adhesive failure (a). Resinous material is evident on the enamel surface conditioned with phosphoric and polyacrylic acid regardless of adhesive action, characterising the mixed failure (b to e).

**Fig 3:**
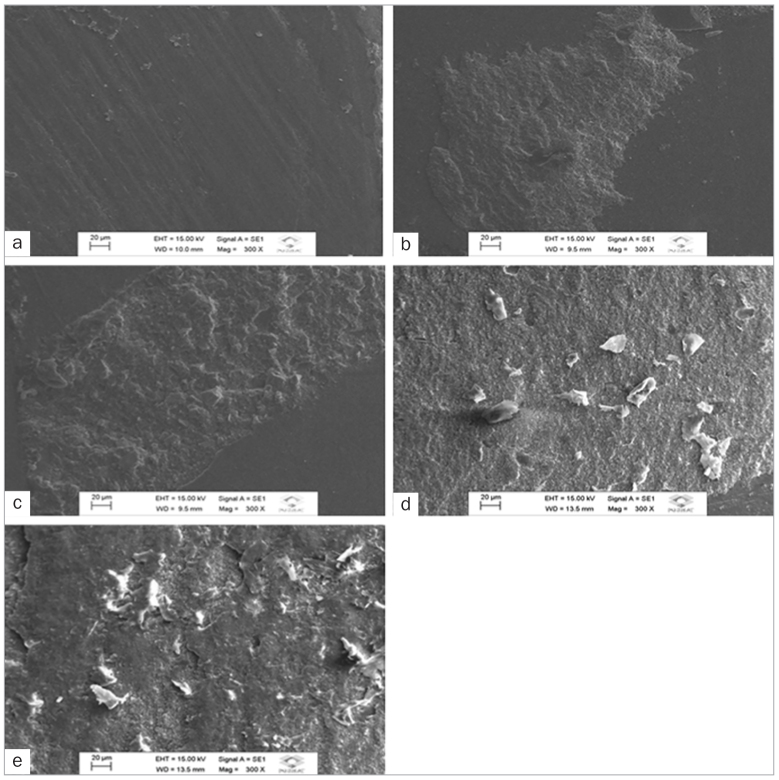
Scanning electron micrographs of representative specimens (original magnification 300X). a. Adhesive failure of dental enamel with no surface treatment luted with MaxCem Elite self-adhesive resin cement (CG/Max group). b. Mixed failure of dental enamel etched with 37% phosphoric acid (FA/Max group). c. Mixed failure of dental enamel etched with 20% polyacrylic acid (PA/Max group). d. Mixed failure of dental enamel etched with 37% phosphoric acid and luted with OptiBond All-In-One dental adhesive and MaxCem Elite self-adhesive resin cement (FA/OB/Max group). e. Mixed failure of dental enamel etched with 20% polyacrylic acid and luted with OptiBond All-In-One dental adhesive and MaxCem Elite self-adhesive resin cement (PA/OB/Max group). Little or no resin cement was observed on enamel surfaces with no surface conditioning, characterising adhesive failure (a). Resinous material was observed on the enamel surface conditioned with phosphoric and polyacrylic acid regardless of adhesive action, characterising mixed failure (b – e).

The results of 2-way ANOVA for microtensile bond strength are shown in Table 2. Table 3 indicated no differences among the self-adhesive resin cements for all groups, except for the group in which the enamel was conditioned with polyacrylic acid. In this group, MaxCem Elite showed higher microtensile bond strength (17.10 ± 3.91 MPa) than did RelyX U200 (9.95 ± 0.87 MPa; p = 0.001) (Table 3). For both self-adhesive resin cements, there were no differences in the bond strength among the groups submitted to different surface treatments independently of the application or not of the dental adhesive for both etching procedures (p > 0.05). Table 4 shows that the RelyX U200 control group had a higher incidence of sticks with premature failure. Figure 1 shows a predominance of mixed-type failure in all evaluated groups, except for the MaxCem Elite control group, which exhibited adhesive failure predominance (Figs 2 and 3). In general, CLSM images showed resin tag formation in the groups subjected to conditioning with phosphoric and polyacrylic acid, independent of the application or not of the adhesive (Figs 5c–e and Figs 6b–e).

**Table 2:** Two-way ANOVA for microtensile bond strengths

Source of variation	df	Sum of squares	Mean square	F	P
Material	1	70.699	70.699	7.427	.0088
Treatment	4	1354.576	338.644	35.574	<.00001
Material x treatment	4	126.714	31.679	3.328	.0171
Residual	50	475.971	9.519		

**Table 3:** Mean ± SD (MPa) microtensile bond strengths as a function of enamel surface treatment and self-adhesive resin cements

	Control group	37% phosphoric acid	20% polyacrylic acid	37% phosphoric acid + dental adhesive	20% polyacrylic acid + dental adhesive
RelyX U200	0.51 ± 0.28^Ab^	13.45 ± 5.22^Aa^	9.95 ± 0.87^Ba^	13.63 ± 1.68^Aa^	13.09 ± 1.42^Aa^
MaxCem Elite	3.10 ± 3.23^Ab^	16.05 ± 4.38^Aa^	17.10 ± 3.91^Aa^	13.05 ± 3.12^Aa^	12.18 ± 2.76^Aa^

Different superscript letters (uppercase in columns, lowercase in rows) indicate statistically significant differences (p < 0.05).

**Table 4:** Number of premature failures as a function of enamel surface treatment and self-adhesive resin cements

	Control group	37% phosphoric acid	20% polyacrylic acid	37% phosphoric acid + dental adhesive	20% polyacrylic acid + dental adhesive
RelyX U200	16	0	0	0	0
MaxCem Elite	4	0	0	0	0

**Fig 4:**
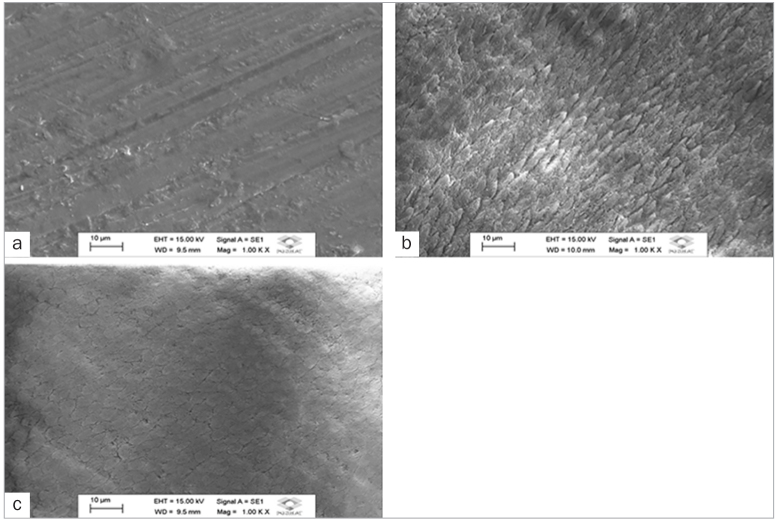
Scanning electron micrographs of enamel surface (original magnification 1000X). a. Enamel surface with no acid etching. b. Enamel surface etched with 37% phosphoric acid. c. Enamel surface etched with 20% polyacrylic acid. Intact enamel surface is evident (a). Homogeneous surface etching removed the smear layer and exposed hydroxylapatite crystals, promoting irregular depths (b) and honeycomb appearance (c) of the surface.

**Fig 5:**
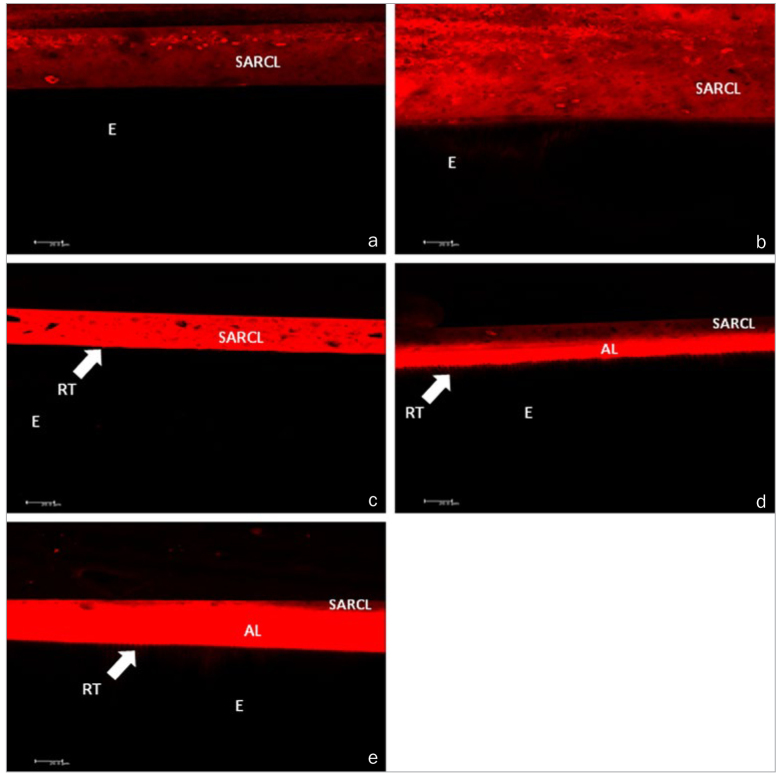
Confocal laser scanning microscopy images. a. Adhesive interface of RelyX U200 self-adhesive resin cement with no enamel surface treatment. b. Adhesive interface of RelyX U200 self-adhesive resin cement on enamel etched with 37% phosphoric acid. c. Adhesive interface of RelyX U200 self-adhesive resin cement on enamel etched with 20% polyacrylic acid. d. Adhesive interface of RelyX U200 self-adhesive resin cement on enamel etched with 37% phosphoric acid and Single Bond Universal dental adhesive. e. Adhesive interface of RelyX U200 self-adhesive resin cement on enamel etched with 20% polyacrylic acid and Single Bond Universal dental adhesive. SARCL, self-adhesive resin cement layer; AL, adhesive layer; E, enamel; RT, resin tags. (a) and (b): No resin infiltration was observed in either the enamel substrate with no acid conditioning or etched with 37% phosphoric acid. (c): Poor, non-uniform resin infiltration was observed into the enamel substrate conditioned with 20% polyacrylic acid. (d) and (e): Deep, uniform bonding agent penetration was observed on the enamel substrate conditioned with 37% phosphoric acid (d) and 20% polyacrylic acid and Single Bond Universal application (e).

**Fig 6:**
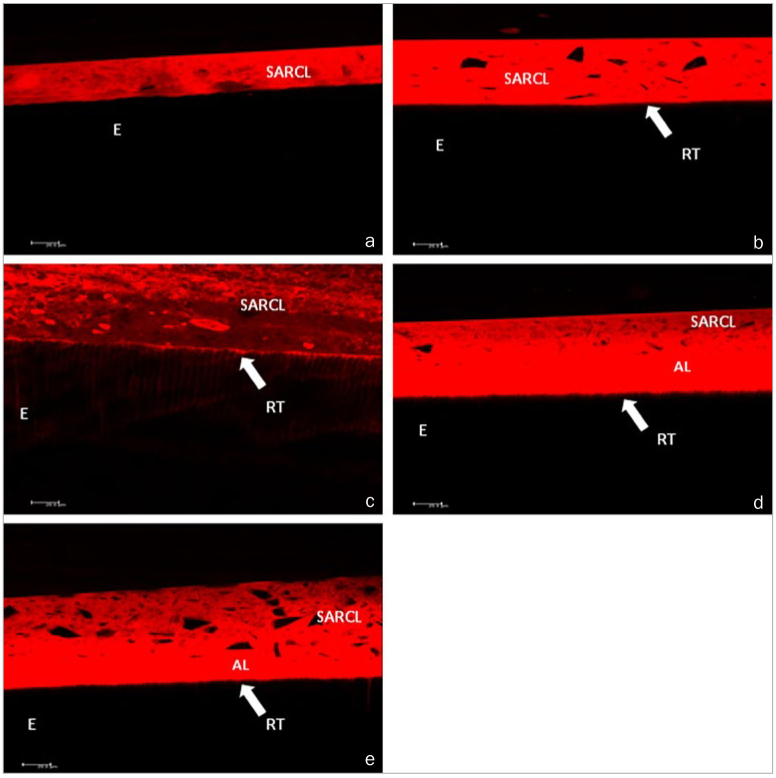
Confocal laser scanning microscopy images. a. Adhesive interface of MaxCem Elite self-adhesive resin cement with no enamel surface treatment. b. Adhesive interface of MaxCem Elite self-adhesive resin cement on enamel etched with 37% phosphoric acid. c. Adhesive interface of MaxCem Elite self-adhesive resin cement on enamel etched with 20% polyacrylic acid. d. Adhesive interface of MaxCem Elite self-adhesive resin cement on enamel etched with 37% phosphoric acid and OptiBond All-In-One dental adhesive. e. Adhesive interface of MaxCem Elite self-adhesive resin cement on enamel etched with 20% polyacrylic acid and OptiBond All-In-One dental adhesive. SARCL, self-adhesive resin cement layer; AL, adhesive layer; E, enamel; RT, resin tags. (a) No resin infiltration was observed into the enamel substrate with no acid conditioning. (b) Poor, non-uniform resin tags were observed in the enamel substrate conditioned with 37% phosphoric acid. (c) to (e) Deep, non-uniform bonding agent penetration was observed on the enamel substrate conditioned with 20% polyacrylic acid (c), with 37% phosphoric acid and OptiBond All-In-One application (d) and 20% polyacrylic acid and OptiBond All-In-One application (e).

## Discussion

Acid pre-conditioning of enamel before bonding influenced the bond strengths of self-adhesive resin cements, leading to rejection of the first null hypothesis. The use of different self-adhesive resin cements resulted in different adhesive bond strengths, so that the second null hypothesis was also rejected.

As the name implies, self-adhesive resin cements do not need prior conditioning of the dentin substrate, because these materials contain phosphorylated monomers.^9,28^ However, according Mushashe et al,^28^ these acidic monomers are unable to promote satisfactory retention on dental enamel when compared to prior conditioning with phosphoric acid. The present findings (Table 3) agreed with those of the previous studies.^9,28^

The bonding of self-adhesive resin cements is based on chemical and mechanical interactions between resin monomers and dental substrate.^28^ Acidic monomers demineralise the substrate, promoting the infiltration of resin particles into interprismatic enamel, resulting in micromechanical retention.^33^ In addition, the functional monomers chemically react with hydroxyapatite crystals on dental enamel, promoting additional retention. However, according to the literature, these interactions are limited to the surface, impairing resin tag formation (Figs 5a and 6a).^28^ The limited action of these resin cements may be attributed to factors such as: 1. their pH, which is about 2.1, and thus too high to promote sufficient enamel etching;^28,30^ 2. higher viscosity which compromises infiltration of the resin particles, leading to short resin tags;^28,32^ and 3. neutralisation due to the water released from the chemical reaction between resin cement and dental enamel, further increasing the pH of the material.^12,28^ Thus, deficient chemomechanical interaction of self-adhesive resin cements on dental enamel (Figs 5a and 6a) results in a less durable adhesive interface, increasing the probability of adhesive failure (Table 3; Figs 2a and 3a).

Conditioning with phosphoric acid removes the enamel smear layer to a depth of 2-7 μm thanks to the low pH of approximately 0.7.^8,36,42^ Polyacrylic acid facilitates smear layer removal, thus increasing the surface contact area. Despite the differences in previous conditioning and dental substrate demineralisation (Fig 4b and 4c), both acid conditioning procedures prior to luting were effective, which showed statistically similar performance (Table 3).^11^ Lack of pre-test failures for both acid conditioning protocols and self-adhesive resin cement confirm the efficacy of adhesion (Table 4), which is corroborated by the SEM images (Figs 2b and 2c, 3b and 3c).

According to some manufacturers, the use of self-adhesive resin cements can be associated with prior acid enamel conditioning and adhesive in order to optimise bond strength. However, the results of the present study showed that bond strength is not dependent on adhesive application (Table 3). It is speculated that acid conditioning increases the efficacy of the acid monomers in self-adhesive resin cements, promoting porosities on the enamel surface (Figs 4b and 4c)^26^ and facilitating the penetration of resin monomers contained in both resin cement and adhesives. This could contribute to the similarity of bond strength between the groups.

Although both resin materials are considered to be self-adhesive resin cements, their behavior differed when the enamel was submitted to prior conditioning with 20% polyacrylic acid (Table 3). It is speculated that this difference is mainly due to the composition of the materials, since MaxCem Elite contains glycero-phosphate dimethacrylate acid (GPDM) (Table 1).^22^ According Han et al,^22^ this material presents low initial pH, and after 48 h, it does not exceed pH 4, enhancing the efficacy of the polyacrylic acid. This fact may have influenced the bond strengths of this resin material compared to RelyX U200, where the initial pH increased to 7 after neutralisation between acid monomers and dental enamel.^24^ This is corroborated by the CLSM images (Figs 5c and 6c) of this study.

According to the adhesion-decalcification concept described by Vieira-Filho et al^43^ and Yoshihara et al,^46^ chemical bonding of functional monomers is dependent on the molecular structures and ionic interaction with hydroxyapatite crystals in enamel. GPDM, a hydrophilic monomer, presents two polymerizable methacrylate groups and one phosphate acid functional group, which could theorically create a stronger polymer network compared to other monomers which present a mono-methacrylate group;^23,46^ this supports the results found in the present study.^43^

The clinical success of oral rehabilitation using ceramic or indirect restorations is directly related to adequate luting and choice of resin luting materials, because these affect the adhesive quality and longevity of the restoration. In this study, prior enamel conditioning with 20% polyacrylic acid yielded bond strengths similar to those obtained with 37% phosphoric acid. In light of these results and those of another study^27^ which found 20% polyacrylic acid to be effective on a dentin substrate, a simplified, effective adhesion protocol for both substrates could be recommend when a self-adhesive resin cement is used for luting indirect restorations. Some limiting factors should be considered, such as the inhomogeneity of the dental substrate and the impossibility of accurately simulating oral cavity conditions in in vitro studies.

## Conclusion

Acid pre-conditioning of enamel with 20% polyacrylic acid yielded bond strength results similar to that of 37% phosphoric acid when self-adhesive resin cements were used.
